# Research Progress on the Chemical Constituents and Pharmacological Effects of *Houttuynia cordata* Thunb and a Predictive Analysis of Quality Markers

**DOI:** 10.3390/cimb47010018

**Published:** 2024-12-31

**Authors:** Zhuo Yang, Peng Ji, Chenchen Li, Fanlin Wu, Yongli Hua, Yanming Wei, Yuxia Cao

**Affiliations:** 1 College of Veterinary Medicine, Gansu Agricultural University, Lanzhou 730070, China; yangz6085@163.com (Z.Y.); lcc1237@163.com (C.L.); huayongli2004@163.com (Y.H.); weiym@gsau.edu.cn (Y.W.); caoyx8049@163.com (Y.C.); 2 Lanzhou Institute of Husbandry and Pharmaceutical Science, Chinese Academy of Agricultural Science, Lanzhou 730070, China; wufl@gsau.edu.cn

**Keywords:** *H. cordata*, chemical constituents, pharmacological action, quality marker, research progress

## Abstract

*Houttuynia cordata* (*H. cordata*) is widely used in respiratory disease control as an important heat-clearing and detoxifying traditional Chinese medicine. It effectively clears away heat and toxins, eliminates carbuncles, and drains pus, and it is diuretic and detoxicating. The aim of this study is to review the botany, chemical composition, pharmacological effects, and quality control of *H. cordata* to establish a better-quality evaluation system. Google Scholar, Baidu Scholar, PubMed, ScienceDirect, Web of Science, and multiple databases, including China National Knowledge Infrastructure (CNKI) and Wanfang Data, were searched. A structural diagram of the compound was drawn using ChemDraw software. *H. cordata* contains volatile oils, flavonoids, and alkaloids. It has antibacterial, anti-inflammatory, antiviral, antioxidant, antitumor, and immunity-enhancing pharmacological effects. By analyzing the literature, it was predicted that Houttuynia sodium, methyl nonyl ketone, quercetin, and quercitrin could be used as the quality markers (Q-marker) of *H. cordata*. This provides a basis for further research into the applications of *H. cordata*.

## 1. Introduction

*Houttuynia cordata* Thunb (*H. cordata*), also known as “Folding Ear Root”, is an important traditional Chinese medicine that exhibits heat-clearing and detoxification. It has been designated as a dual-purpose plant for pharmaceuticals and food by the Ministry of Health of the People’s Republic of China [1]. It was first documented in the “Famous Doctors’ Record” [2]. It is derived from the fresh whole grass or dried aboveground part of *H. cordata*, a plant belonging to the family Houttuyniaceae. *H. cordata* is primarily cultivated in Zhejiang, Jiangsu, Anhui, Hubei, Yunnan, Guizhou, and other regions. It has a pungent, slightly cold taste and is associated with the lung meridian. The compendium of materia medica regards *H. cordata* as an herb that can “dissipate heat and poisonous carbuncles and swellings”. The classification of Herbal Medicinal Properties acknowledges *H. cordata’s* ability to “cure five dips, remove edema, and remove food accumulation”. This paper provides an in-depth discussion of the composition of the chemical constituents and pharmacological effects of *H. cordata*.

The 2020 edition of the Chinese Pharmacopoeia records that it has the functions of clearing heat and detoxification, removing carbuncles and purulent discharge, promoting diuresis, and eliminating dampness. It is widely used in clinical practice to treat phlegm heat, asthma, cough, dysentery, gonorrhea, abscess, and toxic sores. Modern research shows that *H. cordata* has anti-inflammatory, antibacterial, antiviral, antitumor, antioxidant [3], and other pharmacological effects on respiratory, digestive, urinary, and neurological diseases that have clinical efficacy [4]. Depending on various conditions, such as the geographical environment, the time of harvesting, and the extraction method, the chemical composition and content of *H. cordata* vary to a certain extent, and the pharmacological effects also change to different extents; therefore, it is of great importance to study and analyze quality markers (Q-Marker). In 2016, academician Liu Changxia [5] put forward the concept of the Q-Marker, which remedied many deficiencies in the current quality standards of Chinese herbal medicines. A Q-Marker is an indicator substance for the quality control of traditional Chinese medicine that is closely related to the production and processing procedures of Chinese herbal medicines and Chinese medicinal tablets and preparations, as well as the curative effect of traditional Chinese medicine. In this paper, we reviewed and analyzed the literature on the chemical composition and pharmacological effects of *H. cordata* and discussed the theoretical system of Q-Marker in combination with the actual Q-Marker in *H. cordata* to provide a basis for the rational use of *H. cordata* in clinical practice.

Based on this study, the chemical composition, pharmacological effects, and current quality control process of *H. cordata* were reviewed. Under the basic conditions of Q-Marker prediction of traditional Chinese medicine advocated by the academician Liu Changxiao, we carried out prediction and analysis in terms of plant relatedness, the validity of chemical composition, the measurability of chemical composition, quality transfer, traceability, and concoction and processing. This paper presents a summary of the chemical composition of *H. cordata*. According to the structural types of volatile oils, flavonoids, polysaccharides, alkaloids, etc., *H. cordata* can be classified into different types of compounds. The paper analyzes the main pharmacological effects of *H. cordata*, including antibacterial, immunomodulatory, anti-inflammatory, antioxidant, antiviral, antitumor, and anti-pneumonia properties. The analysis is based on the five principles of quality markers as a guide to literature analysis. This paper aims to combine modern scientific and technological means with traditional Chinese medicine to promote the modernization of *H. cordata*.

## 2. Materials and Methods

The literature search was conducted through Google Scholar, Baidu Scholar, PubMed, ScienceDirect, Web of Science, and several databases, including China National Knowledge Infrastructure (CNKI) and Wanfang Data, by searching for keywords such as “*H. cordata*”, “botany”, “chemical composition”, “quality marker prediction”, and “pharmacological effects”.

## 3. Botany

The Houttuyniaceae family comprises only one perennial flowering herbaceous plant species, *H. cordata*, which is native to China, Japan, North Korea, and Southeast Asia. This species is primarily distributed in the provinces and regions south of Shanxi, Gansu, and the Yangtze River Basin in China. It has a fishy odor when crushed and a slightly astringent taste *H. cordata* is a traditional and effective Chinese herbal medicine in the Chinese Pharmacopoeia. In addition to being eaten as a delicacy in many countries, it has a rich history of medicinal use. It is clinically useful for clearing away heat, detoxification, cough relief, the expectoration of phlegm, and the stimulation of urination *H. cordata* is commonly cultivated as both a leafy and root vegetable and prefers moist and shady habitats [6]. It is traditionally used as a vegetable in many parts of China, such as Sichuan, Guizhou, Yunnan, and Hunan. It is also used as a spice or ingredient in various countries, such as India and Japan. *H. cordata* is categorized into roots, stems, leaves, and flowers. The stems are slender, measuring 15 to 50 cm in height, and the petioles are 3 to 5 cm long, cylindrical, and easily breakable. Both leaves and flowers are borne at the top of the stems, and the flowers are yellowish-green and in spikes, with a flowering period from May to August and a maturity period from July to October [7].

## 4. Chemical Composition

*H. cordata* possesses a complex chemical composition, primarily consisting of volatile oil, flavonoids, alkaloids, polysaccharides, etc. The nutritional content of *H. cordata* also includes proteins, fats, carbohydrates, vitamins, amino acids, inorganic salts, and trace elements. The primary pharmacologically active components of *H. cordata* are volatile oils, polysaccharides, and flavonoids [8,9].

### 4.1. Volatile Oil

**Figure 1:**
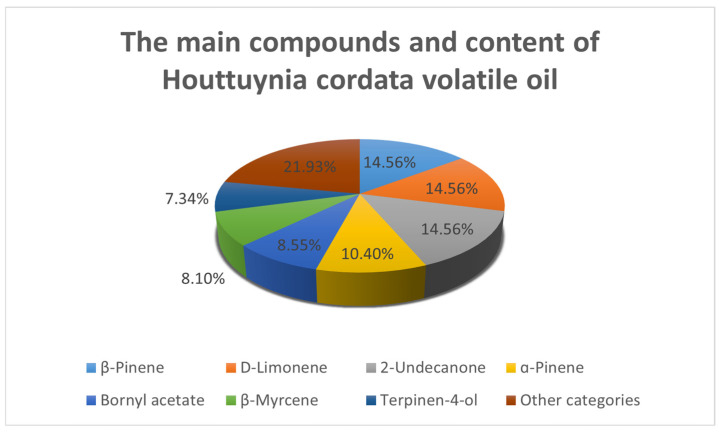
Main compounds and constituents of essential oil of *H. cordata*.

**Figure 2:**
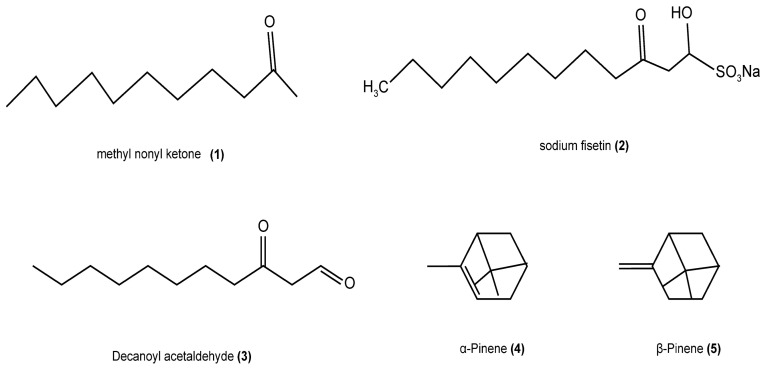
Molecular structures of some compounds.

The *H. cordata* plant contains a volatile oil that constitutes approximately 0.05% of the total composition. The content of the oil varies depending on the growth conditions of the plant, the source of extraction, the time of harvest, and the specific parts utilized and their state. The primary components of this oil include methyl nonyl ketone, decanoylacetaldehyde, and α-pinene. Notably, the volatile oil content varies significantly among different parts of *H. cordata*, with the flower exhibiting the highest concentration, followed by the leaf and stem. This observation is consistent with traditional medicinal practices, which often emphasize the use of the underground parts of the plant. Wei Panpan et al. reported that the classification of the volatile oil of *H. cordata* is shown in Table 1 below [10]. Zhang Yanzhi et al. [11] have reported the main compounds and their contents in the volatile oils of *H. cordata* (Figure 1). Some structures of the compounds have also been described (Figure 2).

#### 4.1.1. Different Extraction Methods

Wu Wenying et al. [12] compared six methods for the extraction of volatile oil from *H. cordata*. They found that the steam distillation method offers a simple extraction process, safe operation, and low cost. In contrast, the supercritical CO_2_ extraction method is characterized by a short extraction time and low operating temperature, which preserves the natural activity of heat-sensitive substances. The decoction method yields a significant amount of water-soluble substances, while the reflux method results in a higher presence of impurities, such as proteins. The maceration method produces a substantial quantity of fat-soluble substances. Additionally, the enzyme-assisted extraction method enhances the yield by incorporating specific enzymes into the steam distillation process. The study concludes that the supercritical CO_2_ extraction method is the most efficient for volatile oil extraction, demonstrating high efficiency, good stability, and attributes such as purity, safety, and non-toxicity.

#### 4.1.2. Different Harvesting Parts

Some studies have revealed disparities in the chemical composition and content of volatile oils among the four parts of *H. cordata*, specifically the root, stem, leaf, and flower. Zhang Shuai et al. [13] utilized GC-MS in combination with chemometrics to identify the volatile constituents and relative abundances in different parts of *H. cordata*. They discovered that 16 categories of 933 volatile compounds were recognized in leaves, aboveground stems, underground stems, and roots. Notably, the underground stems exhibited the highest content of volatile components. The cumulative amounts of 2-undecanone, limonene, and alpha-pinene in the underground stem were significantly greater than those found in the other parts. Additionally, 2-undecanone was found to be a specific active ingredient of *H. cordata*.

#### 4.1.3. Sources of Different Origins

The volatile oil of both wild and artificially cultivated *H. cordata* from various regions exhibits significant differences in composition. Specifically, the volatile oil derived from *H. cordata* in Hunan contains methyl nonyl ketone, 1-nonanol, and decanal. In contrast, the primary components of the volatile oil from artificially cultivated *H. cordata* in Guangzhou include methyl nonyl ketone, decanoic acid, 4-pinene alcohol, and gibberellin acetate. The major components of the volatile oil of *H. cordata* produced in Sichuan Ya’an are not stipulated. The volatile oil of *H. cordata* from the base of *H. cordata* contains methyl nonyl ketone, β-laurene, β-pinene, β-brassicene, decanal, and ɑ-pinene. Song Chenchao et al. conducted a comparative study on the *H. cordata* medicinal materials from three production areas: Boluo County in Guangdong Province, Dali City in Yunnan Province, and Changsha County in Hunan Province. The results showed that the relative concentrations of the four active constituents, namely, (-)-4-terpineol, α-terpineol, sabinene, and β-myrcene, in the volatile oil extracted from *H. cordata* medicinal materials sourced from different production areas showed significant variations. These results suggest that the major constituents of *H. cordata* volatile oils vary by region, which may lead to differences in the efficacy of *H. cordata* sourced from these different areas [14,15,16].

### 4.2. Alkaloids

**Figure 3:**
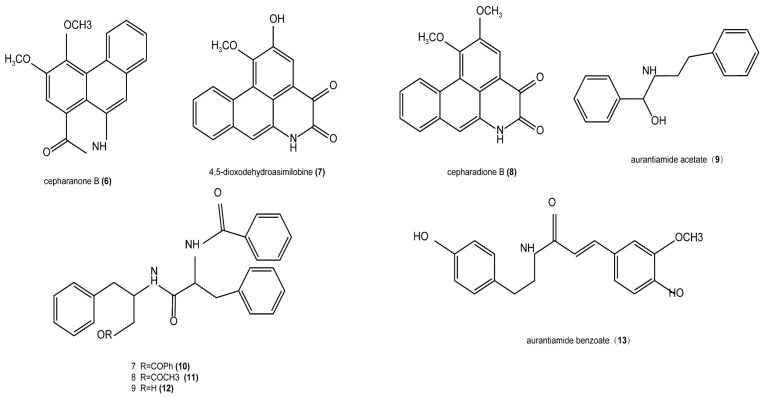
Structures of some alkaloids.

There are 71 compounds of alkaloids reported in *H. cordata*, including aristo lactam, aphophlamide, pyridine, and others; they have biological activities such as antitumor, antiviral, antiplatelet, anti-inflammatory, and hypoglycemic biological activities, and so on [17]. As reported by Chen Shaodan et al. [18], the 11 kinds of alkaloid compounds that were initially isolated from the Houttuyniaceae plants are presented in Table 2, and the structures of some alkaloids are depicted in Figure 3.

### 4.3. Flavonoids

**Figure 4:**
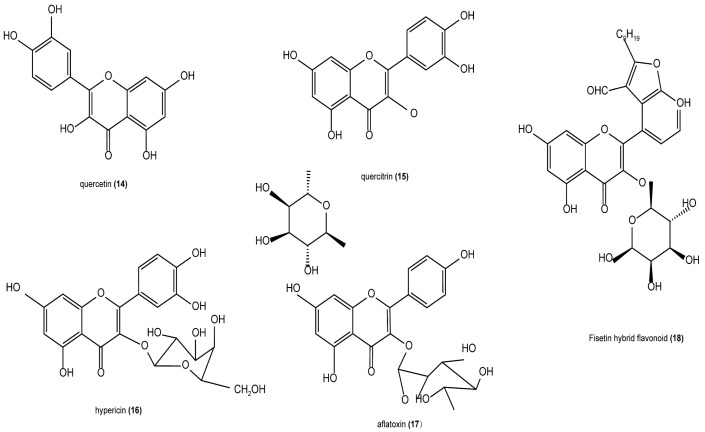
Structures of some flavonoids.

The flavonoid compounds in *H. cordata* account for approximately 0.1% and are one of the main active ingredients of *H. cordata*, including quercetin, quercitrin, isoquercetin, and chrysin [19]. It has also been reported in the literature that most of the flavonoid compounds are in the form of glycosides. The contents of quercetin, isoquercitrin, and rutin in the extract of *H. cordata* are 0.4 μg/mL, 5.4 μg/mL, and 5.4 μg/mL respectively [20]. The structures of the main compounds of *H. cordata* are presented in Figure 4.

### 4.4. Organic Acids

**Figure 5:**
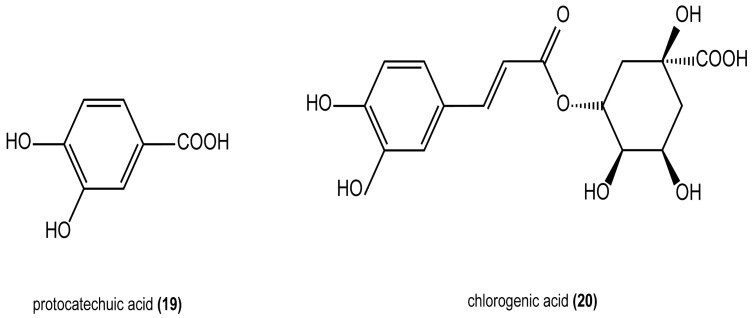
Structures of some organic acids.

Amino acids are important nutrients found in plants. In recent years, some scholars have analyzed the amino acid fractions in *H. cordata*. It contains various organic acids such as chlorogenic acid, palmitic acid, linoleic acid, oleic acid, stearic acid, capric acid, decanoic acid, menadione, aristolochic acid, and octanoic acid [21]. Additionally, 16 types of amino acids, including aspartic acid (Asp), glutamic acid (Glu), serine (Ser), histidine (His), glycine (Gly), leucine (Leu), and isoleucine (Ile), were found in *H. cordata*. The structures of some of the compounds are shown in Figure 5.

### 4.5. Polysaccharides

The pharmacological effects of *H. cordata* can be attributed to polysaccharides, which are large, polar molecules composed of rhamnose, arabinose, glucose, galactose, xylose, mannose, glucuronic acid, and galacturonic acid. Cen et al. also developed a high-purity *H. cordata* polymorpha polysaccharide with an acidic pectin structure and pyranose conformation [22].

### 4.6. Other Constituents

*H. cordata* consists of various chemical components, including proteins, starch, tannins, several salts, like potassium chloride and potassium sulfate, and various vitamins, such as B2, C, P, and E. It also contains metallic elements, such as calcium, magnesium, sodium, and phosphorus, and trace elements such as iron, zinc, manganese, copper, molybdenum, cesium, and so on. The organic acid, amino acid, polysaccharide, and other types of compounds of *H. cordata* are shown in Table 3 [23,24,25,26].

## 5. Pharmacological Effects

The pharmacological effects of *H. cordata* include antibacterial, immunomodulatory, anti-inflammatory, antioxidant, antiviral, antitumor, and anti-pneumonia properties [27,28,29].

### 5.1. Bacteriostatic Effect

**Figure 6:**
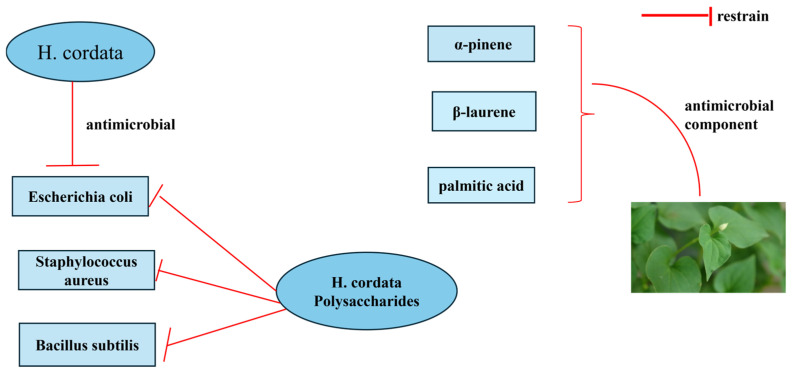
Diagram of inhibitory mechanisms.

*H. cordata* is rich in antimicrobial constituents and has inhibitory effects on a wide range of microorganisms, showing a broad spectrum of bacterial inhibition. Several scholars have discovered that *H. cordata* has bacteriostatic properties against *Escherichia coli*, *Staphylococcus aureus*, and *Bacillus subtilis* when the chemical constituents of *H. cordata* are extracted through hydrodistillation. It has been reported that the crude extract of *H. cordata* contains fifty antimicrobial substances, such as quercetin, protocatechuic acid, dodecanoic acid, ferrous acid, germanic acid, isoquercitrin, butyric acid, undecanoic acid, and so on [30]. In addition, α-pinene, β-laurene, and palmitic acid in the volatile oil of *H. cordata* have been reported to have antibacterial and anti-inflammatory effects. The ethanolic extract of *H. cordata* was found to have antibacterial activity against methicillin-resistant *Staphylococcus aureus* (MRSA) [31]. Polysaccharides are one of the major active components of *H. cordata*. Although the research on the antibacterial effect of the polysaccharides of *H. cordata* is relatively limited in our country, some studies have shown that these polysaccharides can inhibit the growth of seven kinds of food-spoilage bacteria to a certain extent. Furthermore, it has been discovered that different methods of extracting the polysaccharides possess a certain inhibitory effect on *Escherichia coli* and *Staphylococcus aureus* [32]. The mechanism of inhibition is shown in Figure 6.

### 5.2. Anti-Inflammatory Effects

The primary anti-inflammatory active components of *H. cordata* are fisetin and quercetin, which are implicated in the regulation of lymphocyte subpopulations, the inhibition of complement down-regulation, and the modulation of inflammatory signaling pathways and anti-oxidative stress, as well as other mechanisms [33]. At present, decanoylglycolaldehyde, also known as fisetin, is generally considered the main medicinal constituent of *H. cordata* volatilized oils. However, it is unstable and prone to be transformed into methyl nonyl ketone, which has been proven to be one of the constituents exerting an anti-inflammatory effect in volatilized oils. In addition, as an adduct of sodium bisulfite and fisetin, Houttuynia sodium, in addition to the above effects, exerts an anti-inflammatory effect in the treatment of traumatic brain injury [34], cardiac injury [35], pulmonary fibrosis [36], and other diseases.

**Figure 7:**
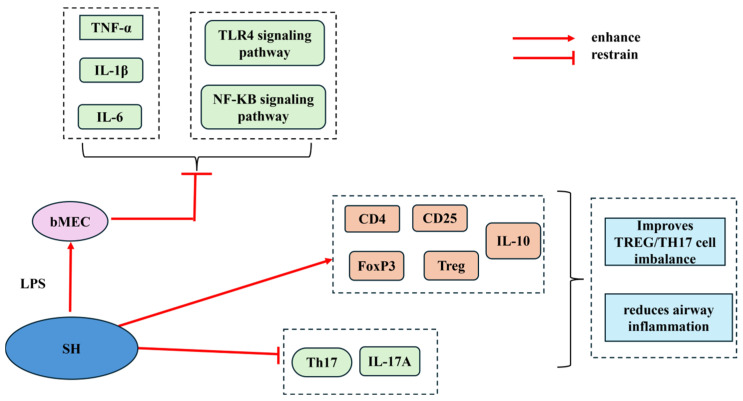
Diagram of the anti-inflammatory mechanism of action of SH.

Some scholars established an experimental mouse model of neutrophilic asthma and intervened with intraperitoneal injection of Houttuynia sodium (SH) or dexamethasone. Subsequently, the degree of lung inflammation was evaluated by collecting bronchoalveolar lavage fluid for cell counting, staining the tissues, detecting cytokine expression by immunohistochemistry, detecting the level of inflammatory factors using ELISA, and analyzing the proportion of the relevant cells in splenocytes using flow cytometry. The results showed that SH improved the Treg/Th17 cell imbalance and reduced airway inflammation by enhancing the frequency of CD4+, CD25+, FoxP3+, and Treg cells and the secretion of the pro-inflammatory cytokine interleukin-10 (IL-10), as well as reducing the generation of Th17 cells and interleukin-17A (IL-17A), which suggests that SH is beneficial for the treatment of asthma [37]. Wang Wenqing et al. [38] similarly reported the antibacterial and anti-inflammatory effectiveness of SH. Through investigating the anti-inflammatory properties of SH on lipopolysaccharide (LPS)-stimulated primary bovine mammary epithelial cells (bMECs), it was found that SH significantly inhibited LPS-stimulated production of tumor necrosis factor-α (TNF-α), interleukin-1beta (IL-1β), and interleukin-6 (IL-6). In Figure 7, the results indicate that SH suppressed the inflammasome’s inflammatory response by inhibiting TLR4 expression and the nuclear factor kappa-B (NF-κB) signaling pathway, indicating SH’s anti-inflammatory action.

**Figure 8:**
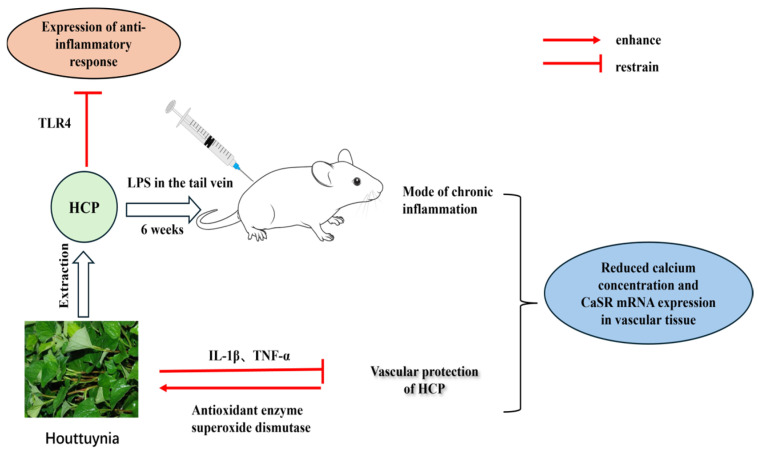
Mechanism of HCP inhibition of LPS-induced chronic vascular inflammation in rats.

Li Teng et al. [39] investigated the activity of the orally administered impact of *H. cordata* polysaccharide (HCP) on vascular chronic inflammation, which was modeled by the tail vein injection of LPS once a week for 6 weeks. The results suggest that HCP can reduce the number of white blood cells and monocytes, repair the histologic damage of vascular tissue, and thus alleviate chronic inflammation. In addition, the protective effect of HCP on blood vessels is also related to the reduction of malondialdehyde (MDA) content, the reduction of IL-1β and TNF-α, and the enhancement of the activities of superoxide dismutase, catalase, and glutathione peroxidase. Meanwhile, HCP exerts anti-inflammatory effects by reducing the expression of toll-like receptor 4 (TLR4) and NF-κB P65, and the combination of HCP and LPS reduces the calcium concentration and CaSR mRNA expression in vascular tissues. These results indicate that HCP inhibits LPS-induced chronic vascular inflammation in rats via calcium-sensitive receptors and the TLR4/NF-κB pathway, and the main mechanisms are shown in Figure 8.

Quercetin, a flavonoid in *H. cordata*, has antioxidant and anti-inflammatory effects. Research has shown that the structural isomer of quercetin, isocoumarin [40], the flavonoid component vitexin [41], the isomer of the flavonoid component rutin, quercetin-3-O-robinobiose [42], and the isomer of hyperoside, isoquercitrin [43], all have anti-inflammatory effects. In addition, 1,5-di-O-caffeoylquinic acid, an isomer of chlorogenic acid, has also been reported to improve dry eye disease by inhibiting inflammation [44]. The abovementioned results indicate that several components of flavonoids all have anti-inflammatory effects. Some scholars have screened methyl nonyl ketone as an anti-inflammatory component of *H. cordata* using an analytical technique combining cell membrane chromatography and GC-MS [36].During the LPS-induced inflammatory response, *H. cordata* polysaccharides, flavonoids, and volatile oil containing 2-undecanone inhibited LPS-induced acute lung injury, ulcerative colitis, and inflammatory response in mice [45,46]. Another researcher found that the alkaloids of *H. cordata* also have anti-inflammatory activity by characterizing the activity of the aboveground parts of the alkaloids [47], and chlorogenic acid and quercetin may be related to the anti-inflammatory properties of *H. cordata* [48]. It has been reported that an aqueous solution of the ethanolic extract of *H. cordata* has bacteriostatic and anti-biofilm effects on oral microorganisms and bacteria and anti-inflammatory effects on oral keratinocytes [49].

### 5.3. Immunomodulatory Effects

*H. cordata* enhances immunity by regulating the complement system of immune cells and immune factors, which can be accomplished by regulating the number of macrophages and T-cells and modulating cytokines [50]. Subsequently, some scholars investigated the aqueous extract of *H. cordata* and discovered that pectic polysaccharides induced the secretion of inflammatory factors, such as IL-1β and TNF-α. These results indicated that the *H. cordata* vulgaris polysaccharides exerted immune effects as an immune-enhancing agent. Similarly, some studies have reported that the fermentation products of *H. cordata* may increase the phagocytic activity of immune cell neutrophils and the proliferation of B-cells, thereby exerting an immune-modulating effect [51].

### 5.4. Antioxidant Effects

Oxidative stress refers to a state of imbalance between oxidation and antioxidants within the body, which is brought about by free radicals in the body. *H. cordata* possesses remarkable antioxidant activity that scavenges free radicals, reduces oxidative stress, and has preventive and therapeutic impacts on a wide range of oxidation-related diseases. Volatile oils, flavonoids, polysaccharides, and polyphenols have been reported to exist in *H. cordata* as potential sources of antioxidants [52,53,54,55]. *H. cordata* contains 16 types of polyphenols, which is an important source of the pharmacological activity of *H. cordata*. The researchers simultaneously determined 16 phenolic substances in *H. cordata* using the HPLC-DAD method, namely, chlorogenic acid, scopoletin, vitexin, rutin, afzelin, isoquercitrin, narirutin, kaempferol-3-O-rutinoside, quercitrin, quercetin, kaempferol, chrysophenol, purple vitexin, hydroxytetramethoxyflavone, hydroxy, tetramethoxyflavone, and camphene alcohol trimethyl ether. Antioxidants, such as polyphenols, effectively scavenge free radicals, preventing living cells from oxidative damage caused by free radicals and slowing aging [56,57,58]. Some scholars have found that the volatile oil and flavonoid constituents of *H. cordata* can scavenge free radicals through the employment of scavenging methods, and they have examined the in vitro antioxidant activity of *H. cordata* by determining the scavenging effect of *H. cordata* volatile oil on hydroxyl and DPPH free radicals [59]. Many components of flavonoids all have antioxidant activity, such as the isomer isocoumarin, which is very important for free radical scavenging activity and shows strong antioxidant activity [60], and there is research suggesting that it is related to the antioxidant activity of isocoumarin through the study of rat colitis [61]. In addition, the flavonoid derivative isoquercitrin and 1,5-di-O-caffeoylquinic acid both demonstrate antioxidant activity [62,63]. Other data have shown that terpenoids in *H. cordata* also have antioxidant mechanisms [64]. Pakyntein Careen Liza et al. [65] proved the antioxidant activity of herbal medicine by conducting anti-inflammatory and antioxidant analyses on the water and alcohol extracts of *H. cordata*. They found that the methanol and water extracts of *H. cordata* have different free radical scavenging and anti-inflammatory activities. The methanol extract of *H. cordata* has higher activity compared to the water extract. Chand et al. [66] reported that the antioxidant activity of *H. cordata*, which mitigated oxidative stress and cellular damage, decreased the level of 1,1-diphenyl-2-picrylhydrazine, and the trivalent iron reduced the antioxidant capacity of the liver and improved the mitochondrial function, thereby protecting the liver against liver damage caused by various external factors.

### 5.5. Antiviral Effect

**Figure 9:**
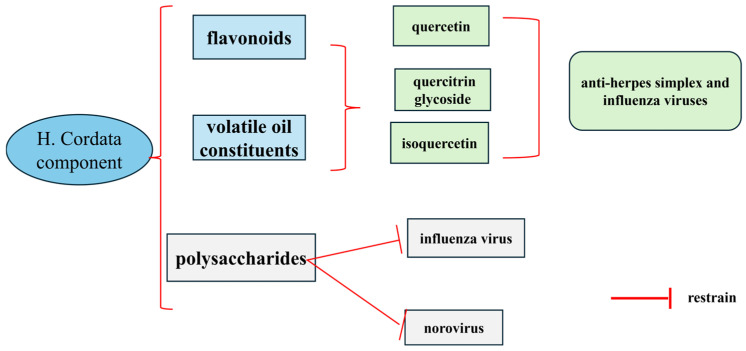
Diagram of the antiviral mechanism.

*H. cordata* is capable of inhibiting the replication of numerous viruses and has therapeutic implications for a variety of viral infectious diseases. It can significantly inhibit a variety of viruses, such as mumps virus, influenza A virus, and influenza B virus, etc. The relevant literature reports that flavonoids and volatile oil constituents have anti-herpes simplex and influenza viruses; aqueous extracts of *H. cordata* and the main constituents, quercetin, quercitrin glycoside, and isoquercetin can inhibit HSV-2 infections by inhibiting NF-κB activation and inhibit HSV-2 infection; and polysaccharide components have an anti-influenza virus and norovirus effect [67,68,69]. Some scholars have antiviral effects through the Houttuynia polysaccharides in the aqueous extracts of *H. cordata* to deform and swell the virus particles, thus inhibiting the penetration of the virus into the target cells, among which the quercetin, quercitrin, and chrysin are the most active. It has been pointed out in the literature that flavonoids and alkaloids in *H. cordata* are non-volatile components, among which Cichlidin hetero-flavonoids have novel structure, strong specificity, and good anti-herpes simplex virus activity [70]. The antiviral mechanism is shown in Figure 9.

### 5.6. Antitumor Effect

Modern pharmacological studies have indicated that the extracts of *H. cordata* have certain antitumor activities, and *H. cordata* has an inhibitory effect on the proliferation of a variety of tumor cells. Wu Xinyu et al. [71] found that the active compounds isolated from *H. cordata* that contribute to the antitumor activity include decanoylacetaldehyde, flavonoids such as quercetin, quercitrin, and rutin, and alkaloids such as aristolactam, isoquinoline, and pyridine. Similarly, it has been reported that flavonoids in *H. cordata* have inhibited cervical cancer [72]. Quercetin has an anti-colon cancer effect; fisetin suppresses 1,2-dimethylhydrazine-induced colon tumorigenesis in Wistar rats by enhancing the apoptotic signaling pathway alkaloids; polysaccharides have inhibited the proliferation of lung cancer cells [73]; and neohesperidin sodium in the volatile oil of *H. cordata* has inhibited tumor growth by inducing apoptosis of HepG2 cells through the mitochondrial pathway, regulated the mRNA expression of the Bcl-2 family, and effectively inhibited liver cancer cells by reducing protein expression and effectively inhibiting the migration of hepatocellular carcinoma cells. Therefore, Houttuynia sodium may be a potential candidate for the treatment of hepatocellular carcinoma [74].

**Figure 10:**
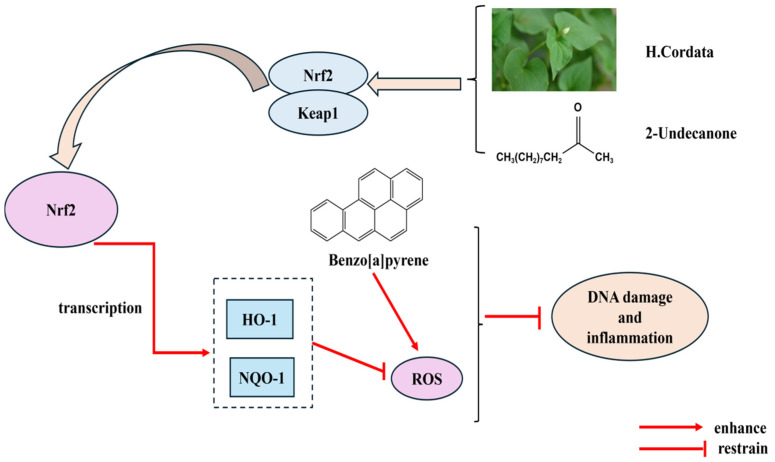
Antitumor mechanism of action diagram.

Lou Yanmei et al. [75] investigated the chemical effects of the bioactive compound 2-undecanone from *H. cordata* sinensis on lung tumorigenesis by establishing an animal model of lung cancer. It was found that *H. cordata* and 2-undecanoate could effectively activate the Nrf2-HO-1/NQO-1 signaling pathway and inhibit the production of intracellular ROS, thus attenuating DNA damage and inflammation induced by Benzo[a]pyrene (B[a]P) stimulation and the results indicated that *H. cordata* and its bioactive compounds also have inhibitory effects on lung tumors, and *H. cordata* is a chemopreventive lung cancer of lung cancer chemoprevention, and its anti-tumor mechanism of action is shown in Figure 10 below. It was found that *H. cordata* and 2-undecanone could effectively activate the Nrf2-HO-1/NQO-1 signaling pathway, inhibit intracellular reactive oxygen species production, and thus attenuate the effects of benzo[a]pyrene (B[a]P) stimulation-induced DNA damage and inflammation; the results indicated that *H. cordata* is chemoprevention for lung cancer and offers protection for B[a]P stimulation-induced DNA damage and inflammation. The results showed that the bioactive compounds of *H. cordata* also inhibited effects on lung tumors and that *H. cordata* is a novel candidate for chemoprevention of lung cancer; the antitumor mechanisms are shown in Figure 10.

### 5.7. Antipneumonic Effects

Viral pneumonia is inflammation of the lungs caused by various viral infections of the upper respiratory tract that spreads downward. It can be transmitted by droplet, contact, the fecal–oral route, etc. The flavonoid and polysaccharide constituents of *H. cordata* are the active components for anti-pneumonia and immunomodulatory functions [76]. Mycoplasma pneumonia is an infectious pneumonia of the respiratory tract caused by Mycoplasma pneumoniae, and some studies have reported that *H. cordata* extract has the effects of inhibiting the release of inflammatory factors, suppressing the activation of inflammatory signaling pathways, and protecting lung tissue in mice with Mycoplasma pneumonia [77,78]. Fewer studies have been reported in recent years on the treatment of bacterial pneumonia with *H. cordata*, but previous studies have reported that *H. cordata* and preparations can have bacteriostatic and bactericidal effects against pneumonia-causing bacteria, such as *Klebsiella*, *Staphylococcus aureus*, *Streptococcus pneumoniae*, and *Streptococcus* spp.

Overall, *H. cordata* contains a considerable amount of nutritional resources and also possesses broad-spectrum anti-inflammatory, antibacterial, and antiviral capabilities. It can enhance the immunity of animals and help prevent and treat a variety of diseases in livestock and poultry. Moreover, it has significant potential for further research and development as a medicinal plant.

## 6. Q-Marker Predictive Analysis

Q-Marker refers to the chemical substances that are inherent in Chinese herbal medicines and Chinese medicine products or that are formed during processing and preparation. They are closely related to the functional properties of Chinese medicines and are used as reference substances to reflect the safety and efficacy of Chinese medicines for quality control purposes. In recent years, the concept of quality markers of traditional Chinese medicine and related research has attracted extensive attention from scholars at home and abroad and has become one of the hot spots of traditional Chinese medicine research. In this paper, based on the concept of quality markers and combined with literature analysis, the Q-Marker of *H. cordata* was predicted to provide a reference for establishing a more scientific quality control approach for *H. cordata*.

### 6.1. Based on Plant Affinity

The Houttuyniaceae, with four genera and six species worldwide, is distributed in East Asia and North America. In China, there are three genera and four species, mainly occurring in the provinces and regions south of central China [79]. *H. cordata* is distributed in the area south of the Yangtze River Basin in China. It contains various chemical components, including volatile oil, flavonoids, and alkaloids. *H. cordata* is the fresh whole grass or the dried aboveground part of *H. cordata* of the Sambucus family, which contains a variety of chemical constituents. There are some differences in content and pharmacological effects, depending on the origin resources, the time of harvest, and the processing methods; therefore, there is a need for a quality evaluation method that can better reflect the uniqueness and overall value of *H. cordata*.

*H. cordata* species generate a wide range of compounds, many of which have antibacterial, anti-inflammatory, antiviral, and antitumor properties [80]. The content of volatile oil in *H. cordata* is plentiful, and the volatile oil, with fisetin as the main active ingredient, is unstable and easily converted to methyl nonyl ketone. It has significant pharmacological activities, such as anti-inflammation and anti-virus activities, and can also be considered as a characteristic component of *H. cordata*. In summary, fisetin and methyl nonyl ketone can be used as candidates for the Q-Marker of *H. cordata* [81].

### 6.2. Validity Based on Chemical Composition

Chinese medicine possesses distinctive medicinal properties and efficacy and shows its effectiveness from different perspectives and levels. These traits are the grounds for the determination of Q-Marker. Modern pharmacological research has revealed that treating diseases with traditional Chinese medicines is mainly based on their medicinal components. Quality control is directed at regulating the efficacy of traditional Chinese medicines.

#### 6.2.1. Correlation Analysis Between Ingredients and Traditional Efficacy

**Figure 11:**
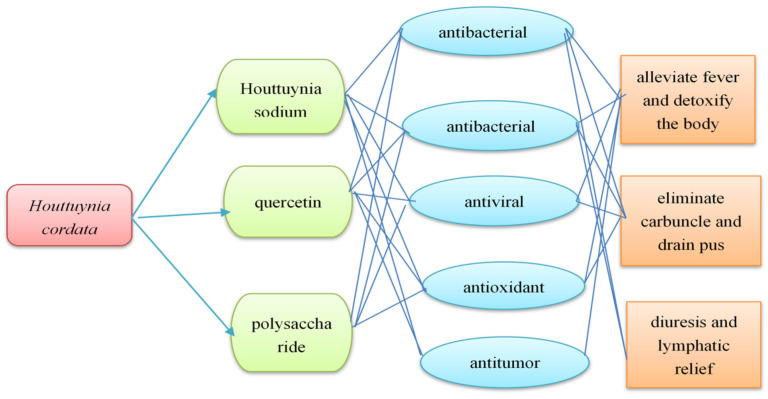
Association diagram of chemical composition and pharmacological action of *Houttuynia cordata*.

Traditional Chinese medicine’s effectiveness is grounded in theoretical foundations. The 2020 edition of the Chinese Pharmacopoeia states that *H. cordata* is effective in clearing heat and toxins, eliminating carbuncles and pus, functioning as a diuretic, and treating phlegm heat, asthma, coughing, hot dysentery, feverish sweating, carbuncles, and swollen and poisonous spores. Modern pharmacological studies have demonstrated that sodium houttuynin in the volatile oil of *H. cordata*, along with the flavonoid components quercetin and polysaccharides, has antibacterial and anti-inflammatory, antiviral, antioxidant, and antitumor pharmacological effects, which are consistent with the traditional efficacy of *H. cordata*. The following figure shows the correlation between chemical constituents and the pharmacological effects of *H. cordata* (Figure 11). These components serve as the primary pharmacological material foundation for the traditional efficacy of *H. cordata* and can be regarded as one of the significant criteria for the screening of quality markers of *H. cordata*.

#### 6.2.2. Correlation Analysis Between Ingredients and Traditional Medicinal Properties

The medicinal properties of traditional Chinese medicine are a highly generalized portrayal of the basic nature and characteristics of the actions of traditional Chinese medicine. They encompass the properties of taste and attribution and are an important basis for traditional Chinese medicine to guide clinical application. Consequently, they can also be taken as a basis for quality markers. *H. cordata* is pungent, slightly cold, and belongs to the lung meridian. Studies have demonstrated that the volatile oil component is the most important “pungent” flavor material basis of traditional Chinese medicine, and the compounds with the highest frequency in cold traditional Chinese medicine are flavonoids, followed by polysaccharides and volatile oils [82]. The highest content of volatile oil is methyl nonyl ketone, which can be used as a quality marker for selection.

#### 6.2.3. Correlation Analysis of Constituents and Modern Medicinal Effects

*H. cordata* is known for its diverse pharmacological effects. The primary pharmacological constituent detected in the volatile oil of *H. cordata* is fisetin, yet it is not stable. In contrast, sodium Houttuyniae is more stable and retains the same activity, making it the more studied constituent in clinical research.

In conclusion, the volatile oil of *H. cordata* possesses anti-inflammatory, antioxidant, and antiviral properties, while the polysaccharide components of *H. cordata* strengthen the immune system. Nevertheless, the complex structure of the polysaccharide substances makes them difficult to purify and extract, making them unfit for use as quality markers. Hence, sodium Houttuyniae, a volatile oil constituent, can be utilized as a preferred Q-Marker [83].

### 6.3. Chemical Composition Measurability

The components for the predictive analysis of quality markers are not only required to be active ingredients but they must also be measurable and biologically active. The chemical substances contained in traditional Chinese medicines are relatively complicated, and it is necessary to clarify their stable and quantitatively determinable substances in order to better establish a quality control evaluation system. Currently, chromatography is the main approach for analyzing the chemical constituents in traditional Chinese medicines. Some scholars have obtained chemical constituents from the leaves of *H. cordata*, specifically the diethyl ether leaf extract, the ethanol leaf extract, and the aqueous leaf extract, which were utilized for the determination of polyphenols and flavonoids in *H. cordata*; the colorimetric method was employed for the quantitative determination of total polyphenols and flavonoids in *H. cordata* extracts. The results indicated that the total polyphenol content varied in different parts of the plant, with the highest content in the stem at 9.4 milligrams per gram and the leaves at 8.2 milligrams per gram and the lowest content in the roots at 5.7 milligrams per gram. Meanwhile, Chen Meixi et al. [57] found that the total polyphenol and flavonoid contents and antioxidant activity of ethanol-extracted *Cichorium* spp. were higher than those of *Cichorium* spp. extracted from distilled water. Some researchers have analyzed the composition of the volatile oil using gas chromatography and GC-MS to determine and analyze *H. cordata*, along with the content of methyl nonyl ketone in the volatile oil [84,85]. There are fewer studies on the determination of the alkaloid content in *H. cordata*, and some scholars have determined the flavonoid components in *H. cordata* by utilizing the high-performance liquid chromatography (HPLC) method to accurately measure the contents of rutin, chrysin, quercetin, and quercitrin [86]. Yang Zhannan et al. [87] identified 16 phenolic compounds, including protocatechuic acid, chlorogenic acid, methyl p-hydroxybenzoate, vanillin, methyl vanillic acid, methyl trans-ferulic acid, proanthocyanidin B, catechins, quinic acid, and fishery sclerotium amide A, in *H. cordata* by using HPLC with photodiode array detection.

In the comprehensive analysis, the volatile oil constituents of *H. cordata*, such as methyl nonyl ketone and Houttuynia sodium, and the flavonoid constituents, quercetin and quercitrin, were closely associated with the efficacy of *H. cordata* on the main pharmacological substance basis and could be determined using GC-MS and HPLC; these constituents can be used as Q-Marker candidates.

### 6.4. Quality Transfer and Traceability

The chemical constituents present in traditional Chinese medicines are the basis for their medicinal effects, and the amounts of these constituents in the medicines can indicate their quality. Therefore, it is crucial to guarantee the quality control of traditional Chinese medicines throughout their entire lifecycles, including growth and synthesis, harvesting and processing, preparation and molding, and entry into the bloodstream.

Some researchers have investigated the metabolites of different parts of *H. cordata* by adopting headspace solid-phase microextraction. By comparing the differential metabolites of the aboveground and underground parts, it was discovered that the most significant medicinal constituent of *H. cordata*, 2-undecanone, was mainly concentrated in the underground part and that most of the major constituents of the underground part had pharmacological effects, such as anti-inflammatory, antibacterial, and antiviral effects [88]. Some scholars have qualitatively detected the content of metabolites in the blood of rats after the injection or oral administration of the volatile oil of *H. cordata* and 2-undecanone by GC-MS. It was found that there were 18 constituents of the volatile oil of *H. cordata* in the oral administration, accounting for 34% of the total constituents, and there were 13 constituents of the volatile oil of *H. cordata* in the injection administration, accounting for 25% of the total constituents. It was also found that there were 17 constituents of 2-undecanone in the oral administration and 15 in the injection administration. These accounted for 24.6% and 35.7% of the total constituents, respectively. The average was 29.8%. This indicates that one-third of them were cometabolites [89].

Jin Hantai et al. [90] used the ultra-performance liquid chromatography-quadrupole-time of flight mass spectrometry coupling technique to identify the components of compound *H. cordata* combinations orally absorbed into the bloodstream. By analyzing the resulting chemical compositions, it was concluded that a total of 15 prototypical components and nine metabolites in plasma, which were mainly flavonoids and organic acids, could be the potential active components of *H. cordata*.

Combining the abovementioned predictive analyses, the volatile oil component methyl nonyl ketone and the flavonoid component 2-undecanone of *H. cordata* can be considered candidates for the Q-Marker.

### 6.5. Different Processing Methods

The therapeutic effect of traditional Chinese medicine is closely related to concoction and processing methods, and different processing methods will have an impact on the content of the internal components of the herbs. Hence, the appropriate concoction and processing methods are of great significance for the stability of the quality of the herbs. Different processing methods affect the contents of the volatile oils and flavonoids of *H. cordata*.

Pan Xue et al. [91] used a GS-MS analysis and chemometrics to make a comparison of the volatile oils of *H. cordata* from two harvest seasons. The dried aerial part of *H. cordata* (DHC) is harvested in summer, while the fresh whole grass of *H. cordata* (FHC) is harvested year-round. The results indicated that the volume of volatile oil recovered throughout the year (FHC) was higher than that recovered in the summer (DHC). The GC-MS fingerprints indicated that the volatile oils recovered from FHC and DHC were generally similar and characterized 69 common chemical constituents, among which the chemical constituents 2-undecanone, β-laurene, and β-pinene had the highest contents. However, there were significant differences in the contents of some constituents, such as α-pinene, β-pinene, 1-nonanol, and geraniol acetate.

It has been reported that flavonoids can be extracted from *H. cordata* using a pressurized liquid extraction method. By controlling the ethanol concentration, solvent rate, temperature, and pressure, it was concluded that a solvent ethanol concentration of 50%, a solvent rate of 1.8 mL/min, a temperature of 70 °C, and a pressure of 8 MPa resulted in a flavonoid yield of 3.152% and a flavonoid content of 23.962%, which were superior to those of the traditional hot immersion and ultrasound-assisted extraction methods [92]. Hung Peiyun et al. studied and reported that the optimal extraction process for total flavonoids in the rhizome of *H. cordata* is an extraction temperature of 60 °C, extraction liquid that is 70% ethanol, a ratio of material to liquid of 1:15, and reflux extraction that is carried out three times at 90 min each. Under these conditions, the yield of total flavonoids in the rhizome of *H. cordata* is 3.637% [70]. Some researchers used HPLC and PDA detection to detect flavonoids in cordyceps, and the results showed that the content of kaempferol was the highest at 0.55%, while the content of kaempferol-3,7,4′-trimethyl ether was the lowest at 0.011% [88].

In conclusion, different mixtures have a remarkable influence on the volatile oil and flavonoid contents of *H. cordata*. These components are highly valuable as they serve as the main medicinal components of *H. cordata*. Therefore, volatile oil and flavonoids can be used as quality markers to determine the potency of the herb. However, more research is needed to explore changes in the specific content of the alkaloid component.

## 7. Conclusions and Prospect

*H. cordata* is a plant with both medicinal and edible values. It contains various kinds of active ingredients; therefore, it exhibits diverse pharmacological characteristics. With the continuous and in-depth exploration of *H. cordata*, more bioactive ingredients will be discovered and utilized, further promoting the development. Therefore, as an important natural ingredient, both the edible value and medicinal value of *H. cordata* deserve our attention. Although many studies have described the pharmacological activity and therapeutic potential of *H. cordata* for a variety of diseases, research on *H. cordata* bioactive compounds needs to be further strengthened. Many of the chemical constituents of *H. cordata* have not been thoroughly investigated. As a high-quality herbal medicine, the market for *H. cordata* is gradually growing. However, the contents of active ingredients in *H. cordata* vary widely among different sources and should be developed and utilized more intensively. In terms of chemical composition, most of the current studies have mainly focused on alkaloids, flavonoids, and polysaccharide compounds, while relatively few studies have been conducted on other active ingredients, such as phenols, terpenoids, and organic acids. Therefore, there is a need to intensify research on the constituents. Regarding quality control, in recent years, chromatography and fingerprinting have emerged as the most common methods for characterizing the chemical composition and quality control of *H. cordata*, but we still need to explore better approaches to make quality control faster and more reliable. A good understanding of the chemical composition of *H. cordata* will not only help to ensure the safety and efficacy of medicinal plants in clinical use but will also help to discover new potentially active compounds and contribute to the expansion of clinical applications based on the similarity of chemical compositions.

Owing to the complexity of the composition of *H. cordata*, there are still many problems in the research, such as a lack of research into the blood components and metabolites of *H. cordata*. Most of the research on *H. cordata* is based on volatile oils, alkaloids, flavonoids, and polysaccharides, while there is less research on the other components. *H. cordata* varies in composition due to the different places of origin, and the research regarding the treatment of diseases with *H. cordata* is mainly centered on animal experiments and cell experiments; therefore, the clinical effect of *H. cordata* in the human body still needs to be further verified. The research on the pharmacodynamic material basis and mechanism of action of *H. cordata* is not thorough enough. At the same time, the inconsistency of *H. cordata* extracting and concocting methods and other factors have led to the varying quality of *H. cordata* on the market. Therefore, it is of great significance to establish a quality evaluation system based on Q-Markers.

Based on the review of the chemical composition and pharmacological effects of *H. cordata*, this article conducts a predictive analysis of the Q-Marker from aspects such as plant phylogeny, the efficacy of the chemical composition, the measurability of the chemical composition, quality transfer and traceability, and processing and preparation. We hypothesized that the Houttuynia sodium, methyl nonyl ketone, quercetin, and quercitrin compounds of *H. cordata* could be used as the Q-Markers for further quality research and provide a scientific basis for the better utilization of *H. cordata*. These compounds can be regarded as Q-Markers for additional quality studies of *H. cordata*, providing a scientific basis for the better utilization of the medicinal resources of *H. cordata*. However, we must also pay attention to the rational use and safety of the veterinary drug *H. cordata* to avoid the adverse effects of abuse and misuse and protect the health and safety of humans and animals.

## Figures and Tables

**Table 1:** Main volatile oil components from *H. cordata*.

No.	Compound Name	Molecular Formula	Ref.
1	decanoyl acetaldehyde	C_12_H_22_O_2_	[10]
2	methyl nonyl ketone	C_11_H_22_O	[10]
3	laurl aldehyde	C_12_H_24_O	[10]
4	decanal	C_10_H_20_O	[10]
5	α-pinene	C_10_H_16_	[10]
6	camphene	C_8_H_16_	[10]
7	β-pinene	C_10_H_16_	[10]
8	β-cinnamene,	C_8_H_8_	[10]
9	lobenyl acetate	C_9_H_10_O_2_	[10]
10	limonene	C_10_H_16_	[10]
11	geranyl	C_13_H_22_O	[10]
12	4-pinoresinol	C_10_H_18_O	[10]
13	nonyl alcohol	C_9_H_20_O	[10]
14	geranyl oxide	C_10_H_16_O_2_	[10]

**Table 2:** Main alkaloid components from *H. cordata*.

No.	Compound Name	Molecular Formula	Ref.
1	cepharanone B	C_17_H_13_NO_3_	[18]
2	piperolactam A	C_16_H_11_NO_3_	[18]
3	aristololactam AII	C_16_H_11_NO_3_	[18]
4	4, 5-dioxodehydroasimilobine	C_17_H_11_NO_4_	[18]
5	norcepharadione B	C_18_H_13_NO_4_	[18]
6	cepharadione B	C_19_H_13_NO_4_	[18]
7	aurantiamide benzoate	C_32_H_30_N_2_O_4_	[18]
8	aurantiamide acetate	C_27_H_28_N_2_O_4_	[18]
9	N-transferuloyltyramine	C_18_H_19_NO_4_	[18]
10	aurantiamide	C_25_H_26_N_2_O_3_	[18]
11	N-phenethylbenzamide	C_15_H_15_NO	[18]

**Table 3:** Other components from *H. cordata*.

No.	Classification	Compound Name	Ref.
1	organic acid	linoleic acid	[24]
2	organic acid	oleic acid	[24]
3	organic acid	palmitic acid	[24]
4	organic acid	octanoic acid	[24]
5	organic acid	aristolochic acid	[24]
6	organic acid	chlorogenic acid	[24]
7	organic acid	aspartic acid	[24]
8	organic acid	glutamine	[24]
9	organic acid	serine	[24]
10	organic acid	histidine	[24]
11	organic acid	glycine	[24]
12	organic acid	leucine	[24]
13	polysaccharide	Water-soluble pectin polysaccharide HCA4S1	[25]
14	polysaccharide	pectin-like acidic polysaccharide	[25]
15	amino acid	Aspartic acid	[25]
16	amino acid	Glycine	[25]
17	amino acid	Glutamine	[25]
18	amino acid	Serine	[25]
19	amino acid	Alanine	[25]
20	amino acid	Histidine	[25]
21	amino acid	Threonine	[25]
22	amino acid	Arginine	[25]
23	amino acid	Tyrosine	[25]
24	amino acid	Valine	[25]
25	amino acid	Methionine	[25]
26	amino acid	Phenylalanine	[25]
27	amino acid	Isoleucine	[25]
28	amino acid	Leucine	[25]
29	amino acid	Lysine	[25]
30	amino acid	Proline	[25]
31	sterols	stigmasterol	[23]
32	sterols	brassicasterol	[23]
33	sterols	β-stitoterol	[23]
34	sterols	spinasterol	[23]
35	sterols	Stigmast-4-en-3,6-dione	[23]
36	triterpenoids	ursolic Acid	[23]
37	triterpenoids	oleanolicacid	[23]
38	triterpenoids	sterculinA	[23]
39	triterpenoids	heptadecanoic acidand tricosanoicacid	[23]
40	inorganic salt	KCl	[25]
41	inorganic salt	K_2_SO_4_	[25]
42	vitamin	Vitamin B2	[25]
43	vitamin	Vitamin C	[25]
44	vitamin	Vitamin P	[25]
45	vitamin	Vitamin E	[25]
46	metal element	sodium	[25]
47	metal element	magnesium	[25]
48	metal element	calcium	[25]
49	metal element	phosphorus	[25]
50	microelement	iron	[25]
51	microelement	zinc	[25]
52	microelement	manganese	[25]
53	microelement	copper	[25]
54	microelement	molybdenum	[25]
55	microelement	cesium	[25]

## Abbreviations

*H. cordata*	*Houttuynia cordata* Thunb
Q-Marker	quality marker
GC-MS	gas chromatography-mass spectrometry
Asp	aspartic acid
Glu	glutamine
Ser	serine
His	histidine
Gly	glycine
Leu	leucine
Ile	isoleucine
MRSA	*Staphylococcus aureus*
SH	*Houttuynia sodium*
IL-10	interleukin-10
bMECs	bovine mammary epithelial cells
HCP	*H. cordata* polysaccharide
LPS	lipopolysaccharide
TLR4	toll-like receptor 4
CMC	cell membrane chromatography
IL-1β	interleukin 1 beta
TNF-α	tumor necrosis alpha
B[a]P	benzo[a]pyrene
HPLC	high-performance liquid chromatography
DHC	dried aerial part of *H. cordata*
FHC	fresh whole grass of *H. cordata*

## References

[1] Tong W., Li M., Sun P., Zeng J., Liu X., Hu Y. (2018). Current status of research on the medicinal and food plant *Houttuynia cordata*. J. Liaoning Univ. Tradit. Chin. Med..

[2] Sun X.S. (2011). Six characteristics and application of dietary therapy to health care in Famous Medical Doctors. New Chin. Med..

[3] Wu Z., Deng X.Y., Hu Q.C., Xiao X.L., Jiang J., Ma X., Wu M.Q. (2021). *Houttuynia cordata* Thunb: An Ethnopharmacological Review. Front. Pharmacol..

[4] Wu Y.X., Ding Q.Y., Liu J., Dai Z., Ma S.C. (2022). Progress of research on chemical composition, pharmacology, and quality control of *Houttuynia cordata*. J. Pharm. Anal..

[5] Liu C.X., Chen S.L., Xiao X.H., Zhang T.J., Hou W.B., Liao M.L. (2016). Quality Marker (Q-Marker) of Traditional Chinese Medicines: A New Concept for Product Quality Control of Traditional Chinese Medicines. Chin. Her. Med..

[6] Liccari F., Boscutti F., Sigura M., Tordoni E., Carpanelli A., Valecic M. (2021). First report of naturalization of *Houttuynia cordata* Thunb. 1783 (Saururaceae) in Italy. Rend. Lincei Sci. Fis. Nat..

[7] Fu J., Dai L., Lin Z., Lu H. (2013). *Houttuynia cordata* Thunb: A Review of Phytochemistry and Pharmacology and Quality Control. Chin. Med..

[8] Ahn J., Kim J. (2016). Chemical constituents from *Houttuynia cordata*. Planta. Med..

[9] Li T., Liu L., Wu H., Chen S., Zhu Q., Gao H. (2017). Anti-herpes simplex virus type 1 activity of Houttuynoid A, a flavonoid from *Houttuynia cordata* Thunb. Antiviral. Res..

[10] Wei P.P., Lou Q., Hou Y., Zhao F.L., Li F., Meng Q.G. (2023). *Houttuynia cordata* Thunb.: A comprehensive review of traditional applications, phytochemistry, pharmacology, and safety. Phytomedicine.

[11] Zhang Y.Z., Liu J.L., Miao J.L., Chen X.B. (2020). GC-MS analysis of the volatile oil composition of the underground part of *Houttuynia cordata*. Chin. J. Ethnomed. Ethnopharm..

[12] Wu W.Y., Li L., Yin S.H., Song Y.H., Li W.J. (2020). Research progress on extraction, composition analysis and application of volatile oil of *Houttuynia cordata*. Food Sci. Technol..

[13] Zhang S., Zhang H.X., Chen S.Q., Yang L., Chen X., Jiang H.Y. (2023). Widely targeted metabolomic deciphers the vertical spatial distribution of flavor substances in *Houttuynia cordata* Thunb. J. Food. Compos. Anal..

[14] Li X.W., He X.R. (2004). Study on the chemical composition of volatile oil of Fritillaria spicata. J. Guangdong. Pharm. Univ..

[15] Song C.C., Yi L.Z., Liang Y.Z. (2013). Research on the volatile oil components of *Houttuynia cordata* medicinal materials from different producing areas. J. Instrum. Anal..

[16] Yang Y.X., Wu L.H., Zhao B.B., Lin Z.W., Fan Z.M., Ye H. (2022). Chemical Compositions of *Houttuynia cordata* Thunb. Volatile Oil and Its Analogues Attenuate *Staphylococcus aureus* Virulence by Targeting α-Hemolysin. Russ. J. Bioorg. Chem..

[17] Ma Q., Wei R., Wang Z., Liu W., Sang Z., Li Y. (2017). Bioactive alkaloids from the aerial parts of *Houttuynia cordata*. J. Ethnopharmacol..

[18] Chen S.D., Gao H., Lu C.J., Zhao R.Z., Yao X.S. (2013). Study on the alkaloids and amide components in *Houttuynia cordata* Thunb. J. Shenyang Pharm. Univ..

[19] Liu X.C., Tian J., Zhou Z.R., Pan Y.Z., Li Z.Q. (2023). Antioxidant activity and interactions between whey protein and polysaccharides from different parts of *Houttuynia cordata*. Front. Nutr..

[20] Song H.W., Shen T., Wu J.C., Lin S.K., Diao E.J., Li Z.P. (2021). Extraction and activity of chemical constituents from *Houttuynia cordata* Thunb by ultrasonic method. Cell Mol. Biol..

[21] Zhong Z.H., Du W.F., Luo Y.Y., Liu J.N., Yang Y.X., Chen H.J. (2023). Compositional analysis of flavonoids, phenolic acids, nucleosides, and amino acids from different parts of Cichlidium fischeri based on UHPLC-QTRAP-MS/MS. Chin. J. Pharm..

[22] Cen L.F., Yi T., Hao Y.Z., Shi C.C., Shi X.L., Lu Y. (2022). *Houttuynia cordata* polysaccharides alleviate ulcerative colitis by restoring intestinal homeostasis. Chin. J. Nat. Med..

[23] Bai L., Ma M.Y. (2018). An overview of the research on *Houttuynia cordata*. J. Xinjiang Prod. Const. Corps. Med..

[24] Liang M.H. (2019). Research on the Chemical Constituents and Pharmacological Effects of *Houttuynia cordata*. Guide Chin. Med..

[25] Lu X.S., Lin Y., Tang L., Zhang T., Hu S.T., Zhang E.B. (2021). Research progress on chemical constituents and safety of *Houttuynia cordata*. Chin. Arch. Trad. Chin. Med..

[26] Zheng Y.J., Peng Q.S., Ma Y.Q., Yang M. (2017). Research progress on the chemical constituents of *Houttuynia cordata* Thunb. Guangdong Chem. Indu..

[27] Hee L.J., Jongmin A., Woong K.J., Gook L.S., Pyo K.H. (2015). Flavonoids from the aerial parts of *Houttuynia cordata* attenuate lung inflammation in mice. Arch. Pharm. Res..

[28] Li J.J., Chen G.D., Fan H.X., Hu D., Zhou Z.Q., Lan K.H. (2017). Houttuynoid M, an Anti-HSV Active Houttuynoid from *Houttuynia cordata* Featuring a Bis-houttuynin Chain Tethered to a Flavonoid Core. J. Nat. Prod..

[29] Li W.B., Yang F., Zhan H.L., Liu B.L., Cai J.R., Lou Y. (2020). *Houttuynia cordata* Extract Ameliorates Bladder Damage and Improves Bladder Symptoms via Anti-Inflammatory Effect in Rats with Interstitial Cystitis. Evid. Base. Complement. Altern. Med..

[30] Chang Y.H., Xia S.W., Fei P., Feng H.X., Fan F.Y., Liu Y. (2023). *Houttuynia cordata* Thunb. crude extract inactivates Cronobacter sakazakii: Antibacterial components, antibacterial mechanism, and application as a natural disinfectant. Food. Control..

[31] Yasuko S., Keiji M., Hiromichi Y., Hiroyuki M., Takashi A., Satoshi O. (2016). Anti-bacterial and anti-inflammatory effects of ethanol extract from *Houttuynia cordata* poultice. Biosci. Biotechnol. Biochem..

[32] Ji Y., Yang J., Yu M., Cao Y., Guo S., Qiao A. (2017). Study on Medicinal Plant Active Substances Extraction and Antibacterial Activity of *Houttuynia cordata*. IOP Conf. Ser. Earth Environ. Sci..

[33] Mai M.L., Yu L.Z., Liu J.S. (2018). Research on the anti-inflammatory effect of “Chinese medicine antibiotic” Fritillariae chinensis and the progress of the clinical application. Pharmacol. Clin. Chin. Mater. Clin. Med..

[34] Yao X.L., Wang S.B., Chen Y.C., Sheng L.Q., Li H.H., You H.C. (2021). Sodium houttuyfonate attenuates neurological defects after traumatic brain injury in mice via inhibiting NLRP3 inflammasomes. J. Biochem. Mol. Toxicol..

[35] Yao P.A., Wei K.Z., Feng J.H., Liu X.N., Xu X., Cui H.Y. (2022). Sodium houttuyfonate protects against cardiac injury by regulating cardiac energy metabolism in diabetic rats. Eur. J. Pharmacol..

[36] Hui S.Y., Han C.M., Yu L.X., Wei Z.D., Jian G. (2021). Sodium Houttuyfonate Inhibits Bleomycin Induced Pulmonary Fibrosis in Mice. Front. Pharmacol..

[37] Wang C., Huang C.F., Li M. (2022). Sodium houttuynia alleviates airway inflammation in asthmatic mice by regulating FoxP3/RORγT expression and reversing Treg/Th17 cell imbalance. Int. Immunopharmacol..

[38] Wang W.Q., Hu X.Y., Shen P., Zhang N.S., Fu Y.H. (2017). Sodium houttuyfonate inhibits LPS-induced inflammatory response via suppressing TLR4/NF-ĸB signaling pathway in bovine mammary epithelial cells. Microb. Pathog..

[39] Li T., Sang T., Song Y.H., Hu X.J., Wu Q., Yao Y.F. (2022). *Houttuynia cordata* polysaccharide alleviates chronic vascular inflammation by suppressing calcium-sensing receptor in rats. J. Funct. Foods..

[40] Stasi L.C.D., Camuesco D., Nieto A., Vilegas W., Zarzuelo A., Galvez J. (2004). Intestinal anti-inflammatory activity of paepalantine, an isocoumarin isolated from the capitula of *Paepalanthus bromelioides*, in the trinitrobenzenesulphonic acid model of rat colitis. Planta Med..

[41] Li S., Liang T., Zhang Y., Huang K., Yang S., Lv H.Y., Chen Y., Zhang C.H., Guan X. (2021). Vitexin alleviates high-fat diet induced brain oxidative stress and inflammation via anti-oxidant, anti-inflammatory and gut microbiota modulating properties. Free Radic. Biol. Med..

[42] Lee S.H., Park H.S., Notsu Y., Ban H.S., Kim Y.P., Ishihara K., Hirasawa N., Jung S.H., Lee Y.S., Lim S.S. (2008). Effects of hyperin, isoquercitrin and quercetin on lipopolysaccharide-induced nitrite production in rat peritoneal macrophages. Phytother. Res..

[43] Liang S., Xu Z., Ruan Y., Niu T.L., Guo W., Jiang W., Hou J.Q. (2020). Isoquercitrin Attenuates Renal Ische-mia/Reperfusion Injury Through Antioxidation, Anti-inflammation, and Antiapop-tosis in Mice. Transplant. Proc..

[44] Yoon C.H., Jang H.J., Ryu J.S., Ko J.H., Ahn K.S., Oh S.R., Oh J.H., Chung J.H., Oh J.Y. (2023). 1,5-Dicaffeoylquinic acid from *Pseudognaphalium affine* ameliorates dry eye disease via suppression of inflammation and protection of the ocular surface. Ocul. Surf..

[45] Tang J.Y., Zhou L.S., Yang G.Q., Liu Y., Shi X.L., Lu Y. (2023). Therapeutic effects on H1N1-induced pneumonia in mice and intestinal bacteria biotransformation of four main flavonoids from *Houttuynia cordata* Thunb. J. Pharm. Biomed. Anal..

[46] Zhu H., Lu X., Ling L., Li H., Ou Y., Shi X. (2018). *Houttuynia cordata* polysaccharides ameliorate pneumonia severity and intestinal injury in mice with influenza virus infection. J. Ethnopharmacol..

[47] Ahn J., Chae H.S., Chin Y.W., Kim J. (2017). Alkaloids from aerial parts of *Houttuynia cordata* and their anti-inflammatory activity. Bioorg. Med. Chem. Lett..

[48] Ho T.Y., Lu H.Y., Lu G.L., Liao P.Y., Hsiang C.Y. (2023). Analysis of target organs of *Houttuynia cordata*: A study on the anti-inflammatory effect of upper respiratory system. J. Ethnopharmacol..

[49] Yasuko S., Keiji M., Hiromichi Y., Takashi A., Natsumi F., Shohei O. (2016). Preventive Effects of *Houttuynia cordata* Extract for Oral Infectious Diseases. Biomed. Res. Int..

[50] Guo H.L., Xu T., Zhang Q. (2022). Progress in the study of immunologic effect and mechanism of action of *Houttuynia cordata*. Heilongjiang Med..

[51] Cheng B.H., Chan J.Y.W., Chan B.C.L., Lin H.Q., Han X.Q., Zhou X. (2014). Structural characterization and immunomodulatory effect of a polysaccharide HCP-2 from *Houttuynia cordata*. Carbohydr. Polym..

[52] Anwar N.A., Jamain A.N., Ridzwan N., Jumli M.N., Hadi N.A., Rohin M.A.K. (2021). Study on Total Phenolic, Flavonoid and Antioxidant Capacity of Fish Singgang Extracts. J. Pharm. Res. Int..

[53] Jing P.M., Cheon K.K., Sungwook C., Sam K.Y., Sun K.H., Won H.J. (2013). Protective Effect of Fisetin (3,7,3′,4′-Tetrahydroxyflavone) against γ-Irradiation-Induced Oxidative Stress and Cell Damage. Biomol. Ther..

[54] Liu L., Zhang Y.Y., Jiang X., Du B.G., Wang Q., Ma Y.L. (2023). Uncovering nutritional metabolites and candidate genes involved in flavonoid metabolism in *Houttuynia cordata* through combined metabolomic and transcriptomic analyses. Plant. Physiol. Biochem..

[55] Suppawit U., Gulsiri S., Kanoknan K., Khanutsanan W., Jintana S., Thanaset S. (2021). Immunomodulatory Potential of the Industrialized *Houttuynia cordata* Fermentation Product In Vitro and in Wistar Rats. Foods.

[56] Arun B.G., Mohammad A.A., Joongku L., Mohammad A.F., Khalid M.A., Fahad A. (2021). Identification of SARS-CoV-2 inhibitors from extracts of *Houttuynia cordata* Thunb. Saudi. J. Biol. Sci..

[57] Chen M.X., Kaiser H.M., Soo K.I., Soon L.J. (2023). Characterization of antioxidant *Houttuynia cordata* extracts loaded polyurethane nanofibers. Fash. Text..

[58] Yang Z.N. (2013). Research on the Correlation Between the Metabolic Accumulation of Phenolic and Volatile Substances in *Houttuynia cordata* and Soil Environmental Factors.

[59] Ju L.L., Zhang J.X., Wang F.J., Zhu D.Q., Pei T.T., He Z.E. (2021). Chemical profiling of *Houttuynia cordata* Thunb. by UPLC-Q-TOF-MS and analysis of its antioxidant activity in C2C12 cells. J. Pharm. Biomed. Anal..

[60] Tianpanich K., Prachya S., Wiyakrutta S., Mahidol C., Ruchirawat S., Kittakoop P. (2011). Radical scavenging and antioxidant activities of isocoumarins and a phthalide from the endophytic fungus *Colletotrichum* sp. J. Nat. Prod..

[61] Liaudanskas M., Kubilienė L., Žvikas V., Trumbeckaitė S. (2021). Comparison of Ethanolic and Aqueous-Polyethylenglycolic Propolis Extracts: Chemical Composition and Antioxidant Properties. Evid. Based Complement. Altern. Med..

[62] Li X., Jiang Q., Wang T., Liu J., Chen D. (2016). Comparison of the Antioxidant Effects of Quercitrin and Isoquercitrin: Understanding the Role of the 6″-OH Group. Molecules.

[63] Li X.C., Li K., Xie H., Xie Y.L., Li Y.Y., Zhao X.J., Jiang X.H., Chen D.F. (2018). Antioxidant and Cytoprotective Effects of the Di-O-Caffeoylquinic Acid Family: The Mechanism, Structure-Activity Relationship, and Conformational Effect. Molecules.

[64] Jin Y., Yang L.P., Hu X. (2016). To Disclose the Anti-Infection/Inflammatory Mechanism of Terpenoids from *Houttuynia cordata* in Virtue of Molecular Simulation. Chest.

[65] Pakyntein C.L., Syiem D., Thabah D., Sunn S.E. (2021). Antioxidant, anti-inflammatory and anti-hyperglycemic activity of aqueous and methanolic extract of *Houttuynia cordata*: An in vitro and in vivo study. GSC Bio. Pharm. Sci..

[66] Chand P.L., Shivankar A., Arti G., Kumar C.J., Venkateswara R.C. (2022). Hepatoprotective and Antioxidant Potential of Phenolics-Enriched Fraction of *Anogeissus acuminata* Leaf against Alcohol-Induced Hepatotoxicity in Rats. Med. Sci..

[67] Cheng D., Sun L., Zou S., Chen J., Mao H., Zhang Y. (2019). Antiviral Effects of *Houttuynia cordata* Polysaccharide Extract on Murine Norovirus-1 (MNV-1)—A Human Norovirus Surrogate. Molecules.

[68] Chiow K.H., Phoon M.C., Putti T., Tan B.K.H., Chow V.T. (2016). Evaluation of antiviral activities of *Houttuynia cordata* Thunb. extract, quercetin, quercetrin and cinanserin on murine coronavirus and dengue virus infection. Asian Pac. J. Trop. Med..

[69] Hung P.Y., Ho B.C., Lee S.Y. (2015). *Houttuynia cordata* targets the beginning stage of herpes simplex virus infection. PLoS ONE.

[70] Li D., Liu J.P., Han X., Wang Y.F., Wang C.H., Li Z. (2017). Chemical Constituents of the Whole Plants of *Houttuynia cordata*. Chem. Nat. Compd..

[71] Wu X.Y., Li H. (2024). The research progress regarding the antitumor effects and mechanisms of active ingredients in *Houttuynia cordata*. Asian J. Surg..

[72] Chen H.G., Feng X.J., Gao L., Suresh M., Anand P., Faiz A.A. (2021). Inhibiting the PI3K/AKT/mTOR signalling pathway with copper oxide nanoparticles from *Houttuynia cordata* plant: Attenuating the proliferation of cervical cancer cells. Artif. Cells Nanomed. Biotechnol..

[73] Han K., Jin C., Chen H.J., Wang P.P., Yu M., Ding K. (2018). Structural characterization and anti-A549 lung cancer cells bioactivity of a polysaccharide from *Houttuynia cordata*. Int. J. Biol. Macromol..

[74] Yang L., Ji W., Zhong H., Wang L., Zhu X., Zhu J. (2019). Anti-tumor effect of volatile oil from *Houttuynia cordata* Thunb. on HepG2 cells and HepG2 tumor-bearing mice. RSC Adv..

[75] Lou Y.M., Guo Z.Z., Zhu Y.F., Kong M.Y., Zhang R.R., Lu L.L. (2019). *Houttuynia cordata* Thunb. and its bioactive compound 2-undecanone significantly suppress benzo(a)pyrene-induced lung tumorigenesis by activating the Nrf2-HO-1/NQO-1 signaling pathway. J. Exp. Clin. Cancer Res..

[76] Ling L.J., Ren A.Q., Lu Y., Zhang Y.Y., Zhu H.Y., Tu P. (2022). The synergistic effect and mechanisms of flavonoids and polysaccharides from *Houttuynia cordata* on H1N1-induced pneumonia in mice. J. Ethnopharmacol..

[77] Shi C.C., Zhou L.S., Li H., Shi X.L., Zhang Y.Y., Lu Y. (2022). Intestinal microbiota metabolizing *Houttuynia cordata* polysaccharides in H1N1-induced pneumonia mice contributed to Th17/Treg rebalance in the gut-lung axis. Int. J. Biol. Macromol..

[78] Zhang J., Chai L., Liu R.J., Li N., Li Y.F., Min X.X. (2021). Effects of Fritillaria extract on TLR-2/MyD88/NF-κB signaling pathway in Mycoplasma pneumoniae mice. Chin. Mat. Med..

[79] Laldinsangi C. (2022). The therapeutic potential of *Houttuynia cordata*: A current review. Heliyon.

[80] Oh S.Y. (2015). An Effective Quality Control of Pharmacologically Active Volatiles of *Houttuynia cordata* Thunb by Fast Gas Chromatography-Surface Acoustic Wave Sensor. Molecules.

[81] Wang L.R., Dong H.Y., Miao M.S. (2024). Research progress of *Houttuynia cordata* and predictive analysis of its quality markers. New Drugs Clin. Pharm. Trad. Chin. Med..

[82] Chen H., Sun H., Yang X.Y., Zeng S.L., Li Z.X., Sun Y.T. (2016). Study the correlation between traditional Chinese medicines’ cold and heat leveling properties and their chemical composition categories. J. Liaoning Univ. Trad. Chin. Med..

[83] Chen J., Wang W., Shi C., Fang J. (2014). A comparative study of sodium houttuyfonate and 2-undecanone for their in vitro and in vivo anti-inflammatory activities and stabilities. Int. J. Mol. Sci..

[84] Yang L.X., Zhang Y.X., Yi H., Yang H., Zhang Q.W. (2010). Determination of methyl nonyl ketone in volatile oil from herbs of *Houttuynia cordata* by GC-MS. Zhongguo Zhong Yao Za Zhi.

[85] Yang Z.N., Lou S.Q., Ma J., Wu D., Hong L., Yu Z.W. (2016). GC-MS analyses of the volatiles of *Houttuynia cordata* Thunb. Pak. J. Pharm. Sci..

[86] Hien N.M., Ly H.D., Minh D.B., Nghia C.N.T., Huong T.T., Han L.N.T. (2023). RP-HPLC-Based Flavonoid Profiling Accompanied with Multivariate Analysis: An Efficient Approach for Quality Assessment of *Houttuynia cordata* Thunb Leaves and Their Commercial Products. Molecules.

[87] Yang Z.N., Sun Y.M., Lou S.Q., Chen J.W., Yu Z.W., Sun M. (2014). Quality evaluation of *Houttuynia cordata* Thunb by high performance liquid chromatography with photodiode-array detection (HPLC-DAD). Pak. J. Pharm. Sci..

[88] Qi S., Zha L.Y., Peng Y.Z., Lou W., Chen K.L., Li X. (2022). Quality and Metabolomics Analysis of *Houttuynia cordata* Based on HS-SPME/GC-MS. Molecules.

[89] Deng K.W., He F.Y., Shi J.L., Liu W.L., Zhou H., Qiu Y. (2011). Study on network compatibility of metabolisms in vivo rat for volatile oil in houttuyniae herba and 2-undecanone. Zhongguo Zhong Yao Za Zhi.

[90] Jin H.T., Liu J.T., Wang J.F., Zhang H.B., Liu X.Y., He H.H. (2021). UPLC-Q-TOF/MS-based serum medicinal chemistry of compound *Houttuynia cordata* Thunb combinations in rats. Chin. Herb. Med..

[91] Pan X., Li H.Y., Chen D.F., Zheng J.J., Yin L.H., Zou J. (2021). Comparison of Essential Oils of Thunb. from Different Processing Methods and Harvest Seasons Based on GC-MS and Chemometric Analysis. Int. J. Anal. Chem..

[92] Zhang Y., Li S.F., Wu X.W. (2007). Pressurized liquid extraction of flavonoids from *Houttuynia cordata* Thunb. Sep. Purif. Technol..

